# Towards authentic institutional allyship by global health funders

**DOI:** 10.1371/journal.pgph.0003024

**Published:** 2024-03-18

**Authors:** Samuel Oji Oti

**Affiliations:** Global Health Decolonisation Movement in Africa, Nairobi, Kenya; PLOS: Public Library of Science, UNITED STATES; McGill University, CANADA

## Introduction

As debates rage about if and how global health should be decolonized [1,2], this paper focuses on how funders can and should work to reduce power asymmetries in global health. Funders include government agencies, private foundations, international non-governmental organizations (INGOs), or corporate actors that provide financial support, resources, and investments aimed at improving health outcomes on a global scale. Most of these funders are headquartered in the Global North and as has been noted elsewhere, they wield significant power and influence over the field and practice of global health [3,4]. While funders have contributed to historic gains in global health [5], their funding practices can be biased and inequitable [6,7], with a large proportion of funding going to Global North and international partners, rather than communities and Global South institutions. Further, some have argued that the contemporary practices of certain funders mirror their colonial heritage and hence, the need for such funders to be “decolonized” [3].

So, what could a “decolonized” funder look like? This paper posits that such a funder is one that embarks on a journey of authentic institutional allyship. The concept of *authentic allyship* has been described as a profound commitment to supporting marginalized groups, amplifying their voices, and actively working towards equity and justice [8]. Authentic allyship is not merely performative, but it requires a deep understanding of the root, systemic causes of inequality and makes a dedicated effort to create lasting change. Authentic allyship has been proposed to counter mindsets and practices that enable or perpetuate various manifestations of power imbalances in global health [9,10].

Much of what has been written about authentic allyship focuses on individuals. Yet in the context of global health practice, individuals generally operate within institutions. Thus, for authentic allyship to thrive, institutions also ought to embark on the authentic allyship journey. To my knowledge, no framework addresses authentic allyship at the institutional level.

## A framework for authentic institutional allyship

**Fig 1:**
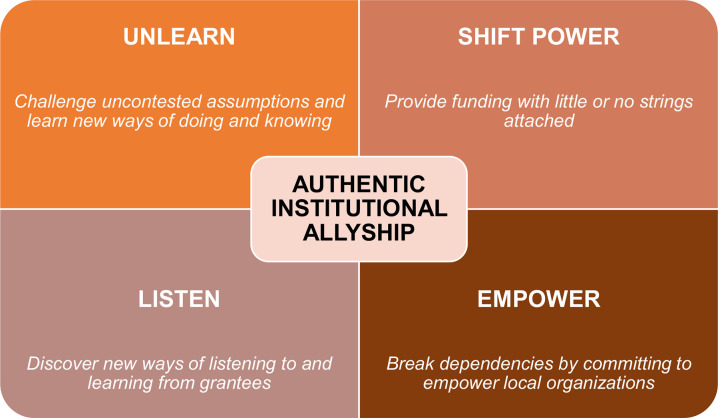
A framework for authentic institutional allyship in global health funding.

In their journey towards authentic institutional allyship, funders must embrace new and different ways of doing business. I propose that funders adopt the following framework that comprises four pillars: 1) Unlearning 2) Shifting power 3) Listening and 4) Empowering (see Fig 1).

### 1. Unlearn

There are several assumptions underlying the policies and practices of funders that enable power imbalances in the practice of global health. For example, funders often cite the lack of capacity in the Global South as a primary reason for preferring to work with "international experts or institutions." This invisibilization of Southern capacity by funders has been described as an “inappropriate generalisation” [7]. Quite often, there is capacity, but funders fail to acknowledge it because they define capacity in ways that elevate elitist intellectualism above indigenous and local knowledge. “Corruption” is another common reason used to primarily fund Global North institutions and researchers, and this assumption assumes that Global North has no corruption (or lobbying, as it is sometimes called). Overall, such harmful assumptions underlie grantmaking practices that often create an undue burden on grantees in the Global South. Unlearning these harmful assumptions will require funders to adopt practices that are more collaborative and less authoritarian. For example, instead of each funder applying their own due diligence procedures to grantees, they could opt for standardized assessments like the Good Financial Grant Practice certification [11].

### 2. Shifting power

Why should a private foundation in the North America or Europe have any say in setting the global health priorities of a country or region in the Global South? Why should such funders have easier access to heads of state and policymakers than grassroots organizations in those countries or regions? Authentic institutional allyship demands the recalibration of power imbalances between funders and their grantees and overturning hierarchical systems of control. Recalibration means that funders willingly shift power by giving out a larger proportion of endowments, unrestricted funding, and core program funding to Global South and community-based organizations − that is, funding with few or no strings attached, and funding with a fair proportion of overhead costs [12]. Philanthropists like MacKenzie Scott have shown that funding without strings attached can be responsibly provided to organizations in the Global South, and that funders can still ensure accountability and fairness while at the same time putting Global South organizations in the driver’s seat [13]. While agencies such as USAID have committed to ‘localization’ progress has been slow due to pushback by INGOs who receive a huge share of USAID contracts.

### 3. Listen

Funders need to develop new ways of listening to and learning from their grantees in the Global South. Traditional methods of receiving feedback such as consultations and third-party evaluations have limitations. Funders should experiment with different approaches. For example, one funder developed a crowdsourcing platform to solicit funding priorities from the public [14]. Funders need to find more dynamic and iterative ways of listening to and learning from their grantees and the communities they aim to serve, so that their funding priorities and practices are more representative and inclusive. Ultimately, every funder’s goal should be to ensure that their grantees in the Global South have significant influence over their funding decisions.

### 4. Empower

Empowerment is a powerful antidote to bias and inequality because it enables individuals, communities and organizations to exert agency over their work or aspirations. The other three pillars of this framework are ultimately geared towards empowering grantees in the Global South. If funders unlearn their biased and inequitable assumptions, if they shift power by giving more favorable funding conditions, and if they truly listen to their grantees in the Global South, then they will enable empowerment. An additional pathway to empowerment is through organizational strengthening. This involves funders providing long-term financial and technical resources to enable organizations in the Global South to strengthen critical capabilities in areas such as financial management, human resources, operations, monitoring and evaluation, among others. Historically, funders have avoided investing in organizational strengthening because such investments do not typically yield immediate or tangible results such as reductions in Disability Adjusted Life Years or mortality. Yet funders must commit long-term to strengthening organizations in the Global South if they sincerely want to address power imbalances in global health. When organizations in the Global South are empowered, they can better define and solve health problems in their contexts, they can renegotiate the rules of engagement with their counterparts in the Global North, and they can even do a better job of holding their own Governments accountable and demanding for more domestic investments in health.

## Conclusion

Authentic institutional allyship is not a destination. It is a journey, and the proposed framework in this paper can help funders to kickstart their journey. Critics of the framework might argue that it is naïve to expect funders to willingly reform themselves. I understand their skepticism, but I am hopeful that a few funders will break the mold and take the bold steps outlined in this paper. Indeed, a few funders are already trying to do things differently. For example, USAID’s renewed commitment to localization includes a desire to “…Shift power to local actors and ensure they have a meaningful seat at the table [15].” I call on other funders to follow suit.
